# Predicting Emergency Department Patient Arrivals at Hospitals Using Machine Learning Techniques

**DOI:** 10.3390/healthcare14091191

**Published:** 2026-04-29

**Authors:** Abdulmajeed M. Alenezi, Mahmoud Sameh, Meshal Aljohani, Hosam Alharbi

**Affiliations:** College of Engineering, Islamic University of Madinah, Madinah 42351, Saudi Arabia; mahmoud.sameh0101@gmail.com (M.S.); mm66332@hotmail.com (M.A.); hsalharbi@iu.edu.sa (H.A.)

**Keywords:** machine learning, forecasting, emergency department, patient arrivals, time series

## Abstract

**Background/Objective:** Emergency Departments (EDs) face persistent challenges with overcrowding, unpredictable patient arrivals, and difficulty forecasting short-term demand. Precise hourly arrival predictions are crucial for effective staffing, optimal resource management, and minimizing entry delays. **Methods:** This paper develops and evaluates a forecasting framework comparing six approaches (a Seasonal Naive baseline, Exponential Smoothing (ETS), Ridge Regression, LightGBM, a hybrid Temporal Convolutional Network (TCN), and a hybrid Long Short-Term Memory (LSTM) network) using de-identified hourly patient arrival records from an ED in Madinah, Saudi Arabia, covering January–November 2024. A set of 183 engineered features is constructed from cyclical time encodings, weekend and public-holiday indicators, structured autoregressive lags, and volatility measures, with all lag-based features verified to use strictly retrospective information. Models are optimized using Bayesian hyperparameter search and trained under an asymmetric loss function that penalizes underprediction to reflect operational risk. **Results:** Results on a 14-day hold-out test set show that Ridge Regression achieves the lowest MAE (3.75, R^2^ = 0.52), with TCN and LSTM essentially tied (MAE 3.80 and 3.85). Diebold–Mariano tests confirm that Ridge, TCN, and LSTM are statistically indistinguishable from one another and that Ridge is marginally significantly better than LightGBM (p=0.028); all four ML models significantly outperform ETS and the Seasonal Naive baseline (p<0.001). On the asymmetric metric, TCN achieves the best AsymRMSE (5.59), reflecting its tendency to err on the safe side of staffing decisions. Robustness is confirmed through sensitivity analysis across penalty factors, feature ablation demonstrating the contribution of each feature group without overfitting, expanding-window cross-validation across three independent monthly test periods, and conformal prediction intervals achieving well-calibrated coverage. **Conclusions:** These results demonstrate that combining engineered temporal features with either a lightweight linear model or a hybrid sequence model yields accurate hourly ED arrival forecasts; whether the achieved accuracy is operationally sufficient for staffing decisions remains a site-specific question that requires clinical validation beyond the scope of this single-center study.

## 1. Introduction

Emergency Departments (EDs) are among the most resource-intensive and unpredictable units within any hospital system. Sudden fluctuations in patient arrivals can overload triage stations, delay treatment, and reduce quality of care [1]. Globally, EDs face recurrent challenges such as overcrowding, long wait times, and staff fatigue, which are often rooted in the inability to anticipate arrival patterns with sufficient temporal precision [2,3]. Traditional scheduling practices rely heavily on historical averages or weekly trends, which fail to account for short-term variability driven by hourly patterns, weekends, public holidays, or abnormal events [4]. Recent advances in machine learning (ML) and artificial intelligence (AI) offer substantial potential for operational planning in emergency medicine. Hourly arrival forecasts can support proactive staff allocation, improved triage efficiency, and reduced entry bottlenecks. Despite a large body of forecasting research in healthcare, most published work targets daily or weekly aggregates, which limits its usefulness for real-time scheduling [4,5]. The few studies that operate at hourly resolution remain limited in scope, and recent comparative work has shown that well-tuned linear models can match or outperform considerably more complex architectures on related daily-level ED tasks [2,6,7]. A detailed treatment of this prior work is given in Section 2.

Three specific gaps motivate the present study. First, despite growing interest in Middle Eastern healthcare AI, including recent Saudi studies on ED data classification [8] and triage frameworks [9], no prior work has developed and validated hourly arrival forecasting models for Saudi Arabian EDs, where Friday–Saturday weekends and Islamic holiday calendars create distinct temporal structures. Second, no study has systematically compared a classical state-space baseline (ETS), a regularized linear model, a gradient-boosted ensemble, and two complementary hybrid deep learning architectures (TCN and LSTM) within a single unified evaluation framework at hourly resolution. Third, asymmetric loss functions that align model optimization with the operational asymmetry between understaffing and overstaffing remain largely unexplored in the ED forecasting literature, despite evidence that prediction intervals alone do not fully address operational risk [2].

The contributions of this paper are therefore:

- A unified six-model hourly forecasting framework (Seasonal Naive, ETS, Ridge, LightGBM, hybrid TCN, and hybrid LSTM) trained and evaluated under a single methodology, including a uniform two-phase training protocol that retrains each iterative model on the full training set after determining its optimal training budget on an internal validation split;
- Application to a previously unstudied Saudi Arabian ED, with feature engineering tailored to the local Friday–Saturday weekend and Islamic holiday calendar;
- An asymmetric mean squared error (AMSE) objective that penalizes underprediction more heavily than overprediction, with a sensitivity analysis across penalty factors p∈[1.0,3.0] confirming the stability of the model ranking;
- A comprehensive validation suite consisting of pairwise Diebold–Mariano tests, bootstrap confidence intervals, a 10-configuration feature ablation, expanding-window cross-validation across three monthly test periods, conformal prediction intervals, and a binned (low/medium/high) tercile evaluation.

## 2. Related Work

### 2.1. ED Overcrowding and the Forecasting Imperative

The operational challenges of Emergency Departments have been a long-standing subject of investigation in health services research. A foundational concern in ED management is overcrowding, which has been linked to adverse patient outcomes including increased mortality, delayed pain management, and higher rates of medical errors. A systematic review of ED overcrowding identified its causes, effects, and proposed solutions, establishing the urgency for data-driven approaches to demand prediction and resource allocation [1]. Early studies on ED demand modeling relied predominantly on classical statistical and time-series techniques. Autoregressive Integrated Moving Average (ARIMA) models were among the first formal approaches applied to patient arrival forecasting, demonstrating the utility of temporal patterns for scheduling. A systematic review of forecasting models for ED visit counts concluded that while simple time-series models capture broad trends, they often fail to account for irregular spikes driven by seasonal illness outbreaks, public holidays, or extreme weather events [4]. This limitation has motivated the development of richer modeling frameworks that incorporate exogenous contextual variables. The influence of environmental and calendar factors on ED demand has been extensively documented. Day of week, time of day, and seasonal factors are consistent and significant predictors of ED patient volumes across diverse hospital systems [10]. Similarly, studies from diverse geographic contexts have confirmed the importance of local cultural calendars including national holidays and weekend structures specific to particular countries in shaping arrival patterns. The comparative value of multivariate versus univariate approaches was further established by Aboagye-Sarfo et al., who showed that multivariate vector-ARMA models provided more precise and accurate forecasts with smaller confidence intervals than univariate ARMA and Winters’ methods [11]. These findings underscore the need for region-specific feature engineering, which directly motivates the Saudi Arabia-specific feature construction in the present study, where Friday and Saturday constitute the weekend and Islamic public holidays represent major structural breaks in arrival patterns. Beyond arrival prediction, general ED research has examined patient flow from entry through discharge, including length of stay, boarding, and diversion. These works show that the ED behaves as a queuing network in which imbalances at any stage, including surges in initial arrivals, propagate downstream to create cascading bottlenecks. Multivariate time-series methods have been applied to jointly model ED demand alongside inpatient hospital resource use, revealing strong temporal relationships between arrivals and downstream capacity constraints [12]. This finding lends strong operational motivation to the forecasting task addressed in the present work.

### 2.2. Machine Learning and Deep Learning for ED Demand Forecasting

The application of AI and ML in Emergency Departments has accelerated considerably over the past decade, spanning three broad domains: demand forecasting [13], clinical triage support [14,15], and resource optimization [13]. Each of these domains has informed the methodological choices made in the present study. Early machine learning applications in the ED focused on decision support at triage, with logistic regression and artificial neural networks applied to predict downstream outcomes such as inpatient admission using routinely collected triage and administrative variables [16]. In the domain of arrival forecasting specifically, a two-step clustering and regression approach was proposed that partitioned historical records into behavioral groups before fitting arrival models, achieving improved accuracy by capturing subpopulation-level dynamics [17]. More recent work has demonstrated that machine learning models with calendar and event-based features consistently outperform classical baselines. Tuominen et al. [7] found that LightGBM and N-BEATS outperformed statistical benchmarks by 9% and 11% respectively for next-day ED occupancy prediction, while Porto and Fogliatto [18] demonstrated that gradient-boosted models with engineered features were the best performers across 11 EDs in three countries for daily arrival forecasting. These results align with the model selection rationale in the present study. Deep learning architectures have been increasingly explored for ED forecasting. Hybrid deep learning frameworks including Long Short-Term Memory (LSTM) networks and autoencoders have been applied to forecast key ED operational features, reporting competitive performance against traditional statistical models [19,20]. However, deep learning models require substantially more data and computational resources to realize their theoretical advantages, a consideration that is especially relevant in single-center studies with limited observation windows such as the one used here. The question of whether complex architectures provide genuine advantages over well-engineered simpler models was directly addressed by Vollmer et al. [6], who found that generalized linear models often matched or outperformed gradient boosting and other complex ML methods for daily ED demand forecasting at two major London hospitals, though model stacking improved overall stability. This finding is particularly relevant for single-center studies with limited training data. Generalized additive models with probabilistic forecasting have also been applied to ED arrivals, demonstrating that uncertainty quantification, not merely point prediction, can directly support staffing decisions by communicating the risk of understaffing under different staffing scenarios [2]. An emerging concern in deployed ED forecasting systems is concept drift: the statistical relationship between features and arrivals shifts over time due to policy changes, pandemics, or evolving population demographics. Susnjak and Maddigan [21] demonstrated that pandemic-induced concept drift degraded forecasting accuracy by over 50%, and that incorporating real-time proxy variables (e.g., Google search trends, public health alert levels) alongside explainable ML analysis could help characterize and mitigate such shifts. This finding motivates the expanding-window validation employed in the present study, which tests whether model performance remains stable across different temporal contexts. Complementary to accuracy, operational trust in ML forecasting requires model interpretability. Pelaez-Rodriguez et al. [22] proposed an explainable ML framework with continuous training for ED visit forecasting, achieving accurate short-term and long-term predictions up to one week while providing interpretable feature importance analysis to support operational decision-making.

### 2.3. AI for Triage and Resource Optimization

In the domain of AI-assisted triage, a growing body of literature has evaluated the use of supervised learning to prioritize and categorize patients at ED entry. A scoping review of AI applications in ED triage, examining 47 studies, found that machine learning-based triage tools generally matched or exceeded the consistency of nurse-assigned triage scores, with particular advantages in handling high patient volumes and complex multi-symptom presentations [23]. Despite promising accuracy, significant barriers to clinical adoption were highlighted, including limited model interpretability, data quality issues, and resistance from clinical staff. These challenges have since driven the development of explainable AI (XAI) methods for healthcare. A broader review of predictive triage models emphasized that ensemble methods such as Random Forests and gradient boosting consistently demonstrated high discriminative performance across diverse ED settings [24]. Resource optimization represents a third pillar of AI application in emergency care. Machine learning has been applied to forecast ED occupancy and related congestion metrics using contextual and operational features, enabling near-term prediction with sufficient lead time to support preemptive interventions such as early discharge planning and bed allocation [7]. Research focused on predicting hospital admission for older ED patients showed that tree-based classifiers significantly outperformed logistic regression in identifying high-risk patients who require early resource commitment [25]. PatientFlowNet, a deep learning framework for patient flow prediction, modeled the full patient trajectory from arrival to disposition using a hybrid recurrent architecture, achieving strong performance on both regression and classification tasks and demonstrating the versatility of deep learning in capturing complex ED dynamics [26,27]. Systematic reviews have increasingly synthesized the body of AI evidence in ED operations. A systematic review of AI applications in hospital admission prediction and flow optimization, covering 42 studies published between 2015 and 2024, found that gradient-boosted tree models and deep learning architectures produced the most consistent improvements across institutional settings, while noting that multi-site generalizability remains a major open challenge [28]. Machine learning applied to early mortality prediction in the ED demonstrated that models trained on admission vitals and triage data could predict in-hospital mortality with AUC values exceeding 0.85, significantly outperforming traditional clinical scoring systems [29].

### 2.4. Regional Context and Generalizability

The majority of ED forecasting studies draw on data from North American, European, or East Asian hospital systems, leaving Middle Eastern contexts underexplored. Recent Saudi Arabian studies have begun to address this gap: Ragab et al. [8] applied machine learning to classify and monitor healthcare data in KSA hospitals, noting the heavy patient inflow driven by events such as Hajj and Umrah, while Menshawi and Hassan [9] developed a triage framework for Saudi EDs using multiple ML paradigms, achieving AUC values of 0.95 for critical care prediction. However, no prior study has developed hourly arrival forecasting models specifically validated for Saudi Arabian ED operations. This gap is significant because Saudi EDs exhibit unique temporal structures (Friday–Saturday weekends, Ramadan effects, and large-scale pilgrimage events) that fundamentally differ from the temporal patterns encoded in Western-trained models.

### 2.5. Summary and Research Gaps

Taken together, the existing literature establishes that machine learning, when combined with carefully engineered contextual features, can substantially improve ED operational forecasting. However, as summarized in Table 1, important gaps remain in regional coverage, model coverage at hourly resolution, and the alignment of training objectives with operational risk. The present study addresses these gaps through a unified six-model comparison at hourly granularity, applied to Saudi Arabian ED data with an asymmetric loss framework.

## 3. System Model and Experimental Setup

This section details our methods of preparing the data and the specific models used, namely, Seasonal Naive, ETS, Ridge, LightGBM, TCN, and LSTM.

### 3.1. Dataset and Feature Engineering

The dataset consists of de-identified patient registration logs from a single Emergency Department in Madinah, Saudi Arabia, spanning from 1 January to 30 November 2024. The raw data was aggregated into an hourly time series representing the total arrivals per hour, yielding 8040 raw hourly observations (335 days × 24 h) and 105,925 total patient arrivals over the 11-month period. The mean arrival rate is 13.2 patients per hour (median 13, standard deviation 5.3, interquartile range 9–17, range 0–36), with the 14-day hold-out test period (17–30 November 2024) exhibiting a somewhat higher mean of 17.1 patients per hour (standard deviation 6.7). After feature engineering, the 723 h that fall within the maximum lag window at the start of the series (approximately 9% of the raw observations) carry undefined lag values and are dropped, leaving 6981 h for training and 336 h for the hold-out test period. To enrich the relatively short observation window, the engineered feature set augments the raw counts with contextual indicators specific to the Saudi setting, including Friday–Saturday weekends and Islamic public holiday markers.

The engineered features encode temporal context at multiple resolutions: hour of the day, day of the week, day of the month, and month of the year, all encoded as sine/cosine pairs to preserve cyclical proximity. Given the strong weekly seasonality observed in the raw series, the autoregressive features were structured rather than dense: each prediction at time *t* has access to the three immediately preceding hours (t−1, t−2, t−3) together with the corresponding 4 h windows at the same time-of-day on each of the previous 30 days. This structured lag design captures both short-range autocorrelation and long-range weekly periodicity while keeping the feature dimensionality manageable. All lag-based features use strictly retrospective data: each feature at prediction time *t* uses only values from t−k with k≥1, so no future information is accessible during training or inference. We verified this property programmatically across all 183 features by computing the minimum temporal offset for each predictor. The composition of the final input features is detailed in Table 2.

### 3.2. Experimental Design and Models

A strict chronological split was employed on the feature-engineered dataset. The period from 1 January to 16 November was used for the training set, with the final 14 days (17–30 November ) held out as the test set for final evaluation. This design ensures that all models are judged on their ability to forecast truly unseen future data.

Six modeling approaches were compared, spanning a classical state-space method, statistical learning, and deep learning, to evaluate which architecture is most effective for this forecasting task:

1. **Seasonal Naive:** A non-parametric baseline forecasting the arrival count to be the same as the count from one week prior.
2. **ETS (Exponential Smoothing):** A Holt–Winters model with additive trend and additive seasonality (seasonal period = 24 h), representing the classical state-space forecasting approach.
3. **Ridge Regression:** A regularized linear model operating on the full engineered feature set, establishing a linear baseline.
4. **LightGBM:** A gradient-boosted decision tree model, known for its high performance on tabular data.
5. **Temporal Convolutional Network (TCN):** A deep learning architecture designed for sequence data, adapted here with a hybrid architecture for multivariate forecasting.
6. **Long Short-Term Memory (LSTM):** A recurrent deep learning architecture, included as an additional deep learning baseline with the same hybrid (sequential + static) input structure as the TCN to ensure a fair sequence-model comparison.

The inclusion of ETS provides a direct comparison with a classical time-series method that does not benefit from feature engineering, addressing the question of whether the engineered features and machine learning models provide genuine value beyond an established statistical baseline.

We establish a baseline for comparison using a seasonal naive forecast. This model operates on the simple assumption that the arrival count for a given hour is identical to the count observed at the same hour in the previous week. This allows us to ensure that more complex models are learning meaningful patterns beyond this strong weekly seasonality.

Formally, let the raw time-series data *D* be represented as the sequence of observations $\left\{y_{1},y_{2},\ldots ,y_{N}\right\}$. The forecast of the naive model at hour *t*, denoted ${\hat{y}}_{t}$, is then defined as

(1) $${\hat{y}}_{t}=y_{t-n}$$

where *n* is the seasonal period, in our case 168 h. This model requires no training or hyperparameter optimization; its performance is evaluated directly on the test set.

To provide a classical time-series baseline, we include ETS (Exponential Smoothing), fitted as a Holt–Winters model with additive trend and additive 24 h seasonality. ETS operates directly on the raw hourly arrival counts without the engineered feature set, providing a comparison between a pure time-series method and the feature-engineered machine learning approaches. Unlike the ML models, ETS captures only temporal autocorrelation and does not incorporate exogenous features such as holidays, weekends, or volatility indicators.

We chose Ridge Regression as a linear baseline to establish the performance ceiling of a model constrained to linear relationships on this feature set. This allowed us to quantify the performance contribution of the complex, non-linear interactions that more advanced models can capture. Unlike standard linear regression, Ridge applies L2 regularization to prevent overfitting and distributes the predictive burden across the provided features, whereas Lasso is better suited to feature selection.

As our primary tree-based model, we selected LightGBM, a state-of-the-art implementation of gradient boosting decision trees. Its primary advantage lies in its ability to model complex, non-linear, and high-order interaction effects between features. For instance, the model can learn that a specific hour of the day has a different predictive weight on a public holiday versus a regular weekday, an interaction that linear models cannot easily capture. We chose LightGBM over other gradient boosting frameworks for its computational efficiency, its ability to handle large feature sets, and its consistent top-tier performance on tabular forecasting tasks in both academic and industrial applications.

To represent the deep learning paradigm, we selected two complementary architectures: a Temporal Convolutional Network (TCN) and a Long Short-Term Memory (LSTM) network. For a fair comparison with the tabular models, both sequence models required access to the same complete feature set. We therefore implemented a hybrid architecture for each, designed to process both sequential and static data concurrently. The hybrid design consists of two parallel input streams: (1) a sequence of raw patient arrival counts spanning the preceding 168 h, which is fed into a sequence-learning backbone (causal dilated convolutions for the TCN, or stacked LSTM cells for the LSTM) to learn temporal dependencies, and (2) a static vector containing the full set of 183 engineered features for the target timestep. The output of each sequence backbone is concatenated with the static feature vector and passed through a common dense head to produce the forecast. This hybrid design ensures information parity across all models, allowing the deep learning architectures to draw on their sequence-learning strengths while still receiving the pre-engineered contextual and autoregressive information available to the tabular models. The TCN and LSTM share an identical training protocol: an 85/15 chronological split of the training set is used during hyperparameter optimization and during Phase 1 of final training for determining the optimal number of epochs; Phase 2 then retrains the chosen architecture on the full training set for the determined number of epochs (see Section 3.3 for the full two-phase protocol).

**Figure 1:**
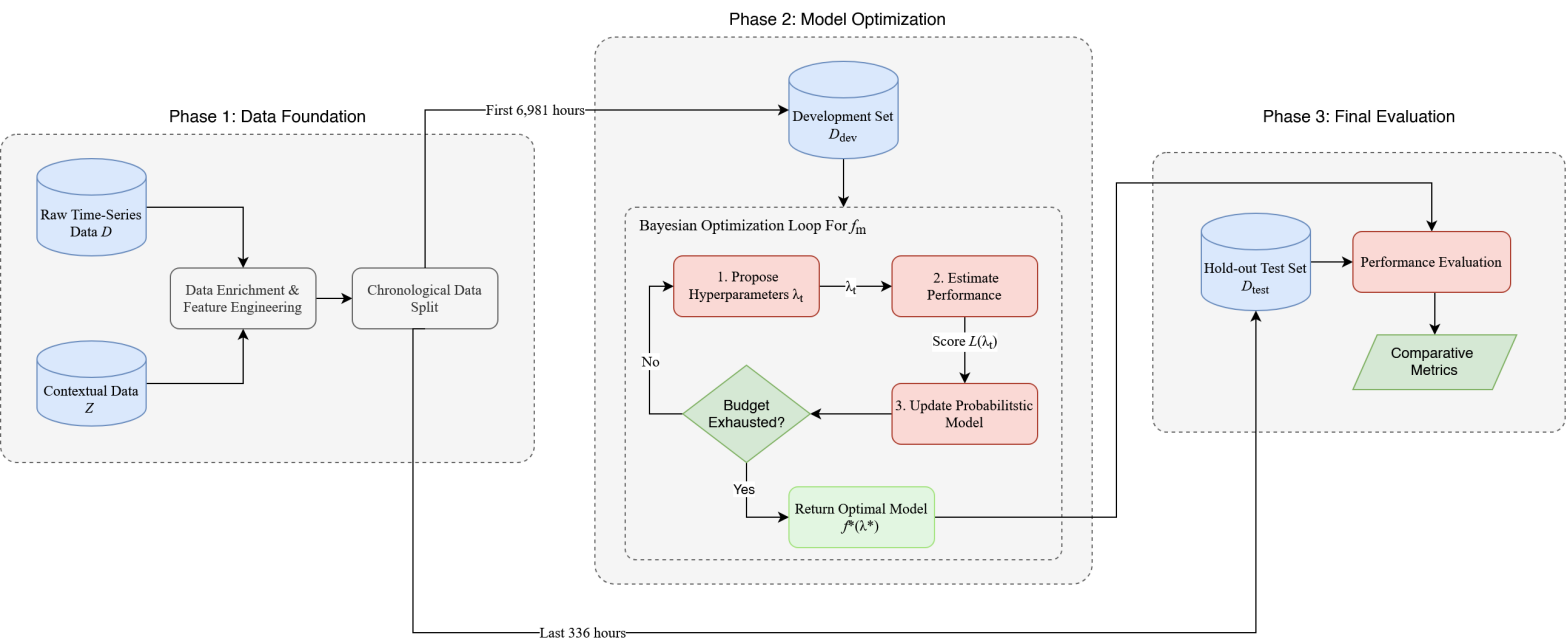
Overview of the experimental methodology. The feature-engineered dataset is chronologically partitioned into a training set (6981 hourly observations) and a final hold-out test set (336 h). The diagram illustrates the general optimization framework applied to each model $f_{m}$. In Phase 2, hyperparameter optimization uses 3-fold time-series cross-validation for the tabular models (Ridge, LightGBM) and a chronological 85/15 split of the training set for the deep learning models (TCN and LSTM), as described in Section 3.3. Final performance for all optimized models is evaluated only once on the unseen hold-out test set to ensure an unbiased comparison.

To systematically evaluate the performance of these approaches, we adopt the unified experimental framework illustrated in Figure 1. The initial raw time-series of hourly arrivals is denoted as the primary dataset *D*. This is enriched with exogenous contextual variables, *Z* (e.g., public holidays and weekend indicators), to form an enriched dataset $D^{\prime }$. This dataset is then chronologically partitioned into a training set, $D_{\mathrm{train}}$, used for all model training and hyperparameter optimization, and a hold-out test set, $D_{\mathrm{test}}$, reserved for final evaluation. Each model, denoted generically $f_{m}$, follows the identical pipeline, which is formalized in Algorithm 1.

### 3.3. Model Optimization and Evaluation

For a fair comparison, the Ridge, LightGBM, TCN, and LSTM models follow a uniform optimization and evaluation protocol. All four models were tuned using Bayesian hyperparameter optimization (Tree-structured Parzen Estimator) with a budget of 100 trials. For the tabular models (Ridge, LightGBM), each trial’s performance was estimated using a 3-fold Time Series Cross-Validation (TSCV) scheme on the training set. For the deep learning models (TCN and LSTM), a single chronological 85/15 hold-out split of the training set was used for HPO trial scoring rather than 3-fold TSCV. This reflects computational constraints: running 100×3=300 full training passes of a deep recurrent or convolutional network is substantially more expensive than the equivalent for tabular models. The single-split compromise for TCN and LSTM was acknowledged and was applied symmetrically to both sequential architectures to preserve fairness of the deep learning comparison.
**Algorithm 1** (End-to-end training and evaluation pipeline for model $f_{m}$). Phase labels correspond to Figure 1.**Require:** Raw time-series *D*, contextual variables *Z*, model class $f_{m}$, search space $\Lambda_{m}$, penalty *P* **Ensure:** Trained model $f_{m}^{*}$, test-set predictions $\hat{y}$, evaluation metrics **  Phase 1: Data Foundation**  1: $D^{\prime }\leftarrow$FeatureEngineer(*D*, *Z*)                        ▹ 183 engineered features  2: $D_{\mathrm{train}},\;D_{\mathrm{test}}\leftarrow$ChronologicalSplit(*D*′)                         ▹ 6981/336 h **  Phase 2: Model Optimization**
  3: Initialize TPE surrogate P(L|λ)  4: **for** t=1 **to** 100 **do**                            ▹ Bayesian HPO, 100 trials  5:     $\lambda_{t}\leftarrow$ProposeSample$\left(P(L|\lambda )\right)$  6:    **if** $f_{m}\in \left\{\mathrm{Ridge},\;\mathrm{LightGBM}\right\}$ **then**  7:         $L\left(\lambda_{t}\right)\leftarrow 3\text{-}\mathrm{Fold}\text{-}\mathrm{TSCV}\left(f_{m},\;D_{\mathrm{train}},\;\lambda_{t},\;{\mathrm{AMSE}}_{P}\right)$  8:    **else**                                       ▹ TCN, LSTM  9:        $L\left(\lambda_{t}\right)\leftarrow {\mathrm{Sin}\mathrm{gleSplit}}_{85/15}\left(f_{m},\;D_{\mathrm{train}},\;\lambda_{t},\;{\mathrm{AMSE}}_{P}\right)$ 10:    **end if** 11:    Update surrogate P(L|λ) with $\left(\lambda_{t},\;L\left(\lambda_{t}\right)\right)$ 12: **end for** 13: $\lambda^{*}\leftarrow \arg\min_{t}L\left(\lambda_{t}\right)$   **Two-Phase Final Training**
14: **if** $f_{m}$ has closed-form solution **then**                              ▹ Ridge 15:    $f_{m}^{*}\leftarrow$ClosedFormfit$\left(D_{\mathrm{train}},\;\alpha^{*}\right)$ 16: **else**                                   ▹ LightGBM, TCN, LSTM 17:    $D_{\mathrm{train}}^{85},\;D_{\mathrm{train}}^{15}\leftarrow$ChronologicalSplit$\left(D_{\mathrm{train}},\;0.85\right)$ 18:    Fit $f_{m}\left(\lambda^{*}\right)$ on $D_{\mathrm{train}}^{85}$ with early stopping on $D_{\mathrm{train}}^{15}$ 19:    $B^{*}\leftarrow$ optimal iteration (LightGBM) or epoch (TCN/LSTM) from early stopping 20:    Reinitialize $f_{m}\left(\lambda^{*}\right)$; retrain on full $D_{\mathrm{train}}$ for exactly $B^{*}$ iterations 21: **end if**   **Phase 3: Final Evaluation**
22: $\hat{y}\leftarrow f_{m}^{*}\left(D_{\mathrm{test}}\right)$ 23: Compute MAE, RMSE, $R^{2}$, AsymRMSE(P), AUCt 24: **return** $f_{m}^{*},\;\hat{y},\;\mathrm{metrics}$

The Bayesian hyperparameter optimization process, which occurs within Phase 2 of our framework (Figure 1), was selected for its superior sample efficiency over exhaustive grid search or random search. The process iteratively builds a probabilistic model, P(L|λ), to approximate the relationship between a given set of hyperparameters, λ, and the model’s performance score, L. At each trial *t*, the algorithm intelligently proposes the next set of hyperparameters, $\lambda_{t}$, that it predicts will yield the most improvement; the score $L(\lambda_{t})$ is then used to update the probabilistic model. After 100 trials, the optimal hyperparameter set, $\lambda^{*}$, is identified. The specific hyperparameters, their functional roles, and the search spaces explored for each model are detailed in Table 3.

Once $\lambda^{*}$ is identified, the final model $f_{m}^{*}$ is trained under a uniform two-phase protocol designed to maximize the use of available training data while preventing overfitting. In Phase 1, the model is fitted on the first 85% of the training set (chronologically), and the remaining 15% is used as an internal validation set to determine the optimal training budget via early stopping: specifically, the optimal number of boosting rounds (for LightGBM) or the optimal number of epochs (for TCN and LSTM). In Phase 2, the model is re-initialized with the same hyperparameters $\lambda^{*}$ and retrained from scratch on the *entire* training set $D_{\mathrm{train}}$ for exactly the training budget identified in Phase 1, with no further early stopping. This protocol ensures that all iterative models use 100% of the training data in their final form, while the training budget is determined solely from an internal validation split that never touches the held-out test set. Ridge Regression, which has a closed-form solution and does not require a validation-based stopping criterion, is fitted directly on the full training set using the single hyperparameter $\alpha^{*}$ selected by HPO.

The best hyperparameters identified by Bayesian optimization for each model are reported in Table 4.

A central component of our experimental design was the choice of the objective function used to guide the optimization process. To align the models with the primary operational goal of minimizing costly underpredictions, all advanced models were uniformly tuned using a custom **asymmetric mean squared error (AMSE)** loss function. This function applies a penalty factor, *P*, to the squared error for any instance where the actual value exceeds the prediction. The AMSE objective is formally defined as

(2) $$\mathrm{Loss}\left(y,\hat{y}\right)=\left\{\begin{array}{cc} {\left(y-\hat{y}\right)}^{2} & \mathrm{if}\;y\leq \hat{y}\;\left(\mathrm{Overprediction}\right)\\ P\cdot {\left(y-\hat{y}\right)}^{2} & \mathrm{if}\;y>\hat{y}\;\left(\mathrm{Underprediction}\right) \end{array}\right.$$

where *y* is the true arrival count, $\hat{y}$ is the model’s prediction, and the penalty factor *P* is set to 2.0. This value reflects the operational judgment that an hour of understaffing from an underprediction is twice as detrimental as an hour of overstaffing from an equivalent overprediction.

The point at which AMSE enters the training pipeline differs across the four advanced models. For LightGBM, AMSE is implemented as a custom objective function with closed-form gradient and Hessian and is therefore optimized directly during boosting. For TCN and LSTM, AMSE is supplied as the Keras loss function and is minimized by the Adam optimizer at every step. Ridge Regression has a closed-form L2 solution that cannot accept an arbitrary loss function; for Ridge, AMSE enters only as the scoring criterion of the Bayesian hyperparameter search, which selects the regularization strength $\alpha^{*}$ that minimizes AMSE on the cross-validation folds. ETS and Seasonal Naive are reported as classical baselines under their standard likelihood-based/persistence formulations and are not exposed to the asymmetric objective.

### 3.4. Implementation Details

To ensure reproducibility and provide transparency regarding our methodology, we document several key implementation details that affect model training and evaluation:

**Data Preprocessing.** The TCN model required MinMax scaling (0–1 normalization) of all features, with the scaler fitted exclusively on the training data to prevent information leakage. Ridge Regression and LightGBM operated directly on the unscaled engineered features, as tree-based models are invariant to monotonic transformations and regularized linear models can handle features of different scales through the regularization parameter.

**Sequence Construction.** For the sequential input stream of the TCN and LSTM, we used a sliding window with a fixed sequence length of 168 h (one full week) to capture the dominant weekly seasonality. At each target timestep, the 168 h historical window of raw arrival counts was paired with the complete static feature vector for that timestep, allowing the deep models to combine sequence-level temporal patterns with the engineered contextual features.

**Reproducibility settings.** All experiments used Python 3.13.5 with a single fixed random seed (42) propagated through NumPy, Python’s random, and TensorFlow. TCN and LSTM were trained with the Adam optimizer at the per-trial learning rate selected by HPO, a batch size of 64, and the AMSE loss defined in Equation (2). The full configuration (hyperparameter search spaces, training budgets, and selected values) is reported in Table 3 and Table 4, and the complete training scripts are provided in the public code release accompanying this paper.

**Early Stopping and Convergence Criteria.** To prevent overfitting and reduce computational cost, we employed early stopping with model-specific patience thresholds. For LightGBM during hyperparameter optimization, training was terminated if validation performance did not improve for 50 consecutive boosting rounds; for the Phase 1 final-training step, this was extended to 100 rounds. For the deep learning models (TCN and LSTM), HPO trials halted after 5 epochs without validation improvement (maximum 15 epochs), and Phase 1 final training used 10 epochs of patience with a maximum of 50 epochs. The optimal training budget identified in Phase 1 (best iteration for LightGBM, best epoch for TCN/LSTM) was then used for the Phase 2 retraining on the full training set, with no further early stopping. Ridge Regression, being a closed-form solution, required no iterative training or early stopping.

### 3.5. Performance Evaluation Metrics

We evaluate each model on one primary, operationally motivated metric and three standard regression metrics, complemented by a binned tercile (Low/Medium/High) classification analysis reported in Section 4.9 that re-expresses the continuous forecasts in terms of the discrete staffing-level decisions they ultimately inform.

1. **Primary Metric: Average Undercount (AUCt).** This metric is designed to quantify the average magnitude of error specifically during underprediction events, which are the most critical from an operational standpoint. It is calculated as the mean of all positive residuals (i.e., instances where the actual count exceeded the forecast). Let *U* be the set of all time indices where an underprediction occurred, such that $U=\{t|y_{t}>{\hat{y}}_{t}\}$. The AUCt is then defined as
  (3) $$\mathrm{AUCt}=\frac{1}{\left|U\right|}\sum_{t\in U}\left(y_{t}-{\hat{y}}_{t}\right)$$
  A lower AUCt indicates a model that, when it does underpredict, does so by a smaller, more manageable margin.
2. **Secondary Metrics.** To ensure a holistic understanding of model behavior, we include the following standard regression metrics:
  - **Mean Absolute Error (MAE):** Provides a clear, interpretable measure of the average absolute prediction error in the original units of the data (patients per hour). It is less sensitive to large, sporadic errors than squared-error metrics:
    (4) $$\mathrm{MAE}=\frac{1}{N}\sum_{t=1}^{N}\left|y_{t}-{\hat{y}}_{t}\right|$$
  - **Root Mean Squared Error (RMSE):** This metric penalizes larger errors more heavily than MAE because residuals are squared. It is particularly relevant here, since large prediction errors (over or under) can have significant operational consequences:
    (5) $$\mathrm{RMSE}=\sqrt{\frac{1}{N}\sum_{t=1}^{N}{\left(y_{t}-{\hat{y}}_{t}\right)}^{2}}$$
  - **R-squared (R^2^):** The coefficient of determination measures the proportion of the variance in the dependent variable that is predictable from the independent variables. It provides a normalized measure of goodness-of-fit:
    (6) $$R^{2}=1-\frac{\sum_{t=1}^{N}{\left(y_{t}-{\hat{y}}_{t}\right)}^{2}}{\sum_{t=1}^{N}{\left(y_{t}-\bar{y}\right)}^{2}}$$
    where *N* is the total number of observations in the test set, $y_{t}$ is the actual value, ${\hat{y}}_{t}$ is the predicted value, and $\bar{y}$ is the mean of the actual values.

The final evaluation of all models is conducted on the hold-out test set ($D_{\mathrm{test}}$) using this full suite of metrics.

## 4. Results

This section presents the comparative performance of the six forecasting approaches on the held-out test set comprising 14 days of unseen data. We report standard regression metrics and operationally motivated asymmetric performance measures, followed by statistical validation, sensitivity analyses, and uncertainty quantification.

### 4.1. Overall Model Performance

Table 5 presents the complete performance profile for all six models. The three top-performing models (Ridge Regression, TCN, and LSTM) are tightly clustered. Ridge Regression achieved the lowest Mean Absolute Error (MAE) at 3.75 patients per hour, with an RMSE of 4.68 and R^2^ of 0.516, indicating that the model explains approximately 52% of the variance in hourly arrivals on the test set. This $R^{2}$ is moderate rather than high, consistent with the substantial intrinsic stochasticity of hour-level ED arrivals; the practical implications of this residual variance are discussed in Section 5.4. TCN is a close second on MAE (3.80) and notably achieves the best Asymmetric RMSE (5.59) and the lowest Average Undercount (AUCt = 3.65). LSTM performs nearly identically to TCN (MAE 3.85, AsymRMSE 5.93).

LightGBM places fourth (MAE 3.94, AsymRMSE 6.35), still significantly better than the classical baselines but marginally trailing the other ML models. ETS achieves an MAE of 4.21, outperforming the Seasonal Naive baseline (MAE 4.89) but falling short of all four feature-engineered ML models.

The Asymmetric RMSE (AsymRMSE), which applies a 2.0× penalty to underpredictions, slightly rearranges the top of the table: TCN (5.59) leads, followed by Ridge (5.86) and LSTM (5.93), then LightGBM (6.35). The classical baselines trail (ETS 6.91, Seasonal Naive 7.96). Ridge achieves a 23.3% MAE reduction and 26.4% AsymRMSE reduction compared to the Seasonal Naive baseline; TCN achieves a 22.3% MAE reduction and 29.8% AsymRMSE reduction.

To assess the reliability of these point estimates, we compute 95% confidence intervals via bootstrap resampling (1000 iterations). Ridge’s MAE has a 95% CI of [3.46, 4.05] and AsymRMSE of [5.40, 6.36]. The TCN MAE CI of [3.53, 4.08] and the LSTM CI of [3.57, 4.14] both substantially overlap with Ridge, consistent with the statistical-test results below showing no significant differences among these three models. The LightGBM CI [3.64, 4.27] is also overlapping but its center is shifted higher, foreshadowing a marginal but statistically detectable gap relative to Ridge. The AsymRMSE CIs do not overlap between any ML model and the classical baselines, confirming clear separation between the modern and classical approaches.

Ridge and LSTM exhibit balanced prediction profiles (both at 50.3% underpredictions), and LightGBM is only mildly imbalanced (54.5% underpredictions). TCN tilts slightly toward overprediction (43.8% under, 56.2% over), consistent with its lowest AsymRMSE: the asymmetric loss penalizes underpredictions, and the TCN training yields a model that errs slightly on the safe side of staffing decisions. ETS shows a pronounced underprediction tendency (58.9%), reflecting the difficulty of fitting a univariate state-space model to data with strong exogenous structure.

### 4.2. Statistical Validation

Table 6 presents pairwise Diebold–Mariano (DM) tests for all 15 model pairs. A clear tiered structure emerges: (1) all four ML models (Ridge, TCN, LSTM, and LightGBM) and ETS significantly outperform the Seasonal Naive baseline (p<0.001); (2) all four ML models significantly outperform ETS: Ridge (p<0.001), TCN (p<0.01), LSTM (p<0.001), and LightGBM (p<0.001); (3) within the ML group, Ridge is marginally significantly better than LightGBM (p=0.028), while the differences among Ridge, TCN, and LSTM are not statistically significant. The differences between LightGBM and TCN/LSTM are also not significant. The top three models therefore form a statistically indistinguishable cluster on the MAE metric, while LightGBM trails this cluster by a small but detectable margin.

These findings were corroborated by non-parametric Wilcoxon signed-rank tests and parametric paired *t*-tests, and by a Friedman test ($\chi^{2}$ significant, p<0.001), confirming overall differences across models.

### 4.3. Sensitivity to Asymmetric Penalty Factor

To justify the choice of penalty factor p=2.0 in the asymmetric loss function, we conducted a sensitivity analysis across p∈{1.0,1.5,2.0,2.5,3.0}, both at evaluation time (recomputing metrics on fixed predictions) and at training time (retraining Ridge and LightGBM with each *p* value as the training objective).

**Figure 2:**
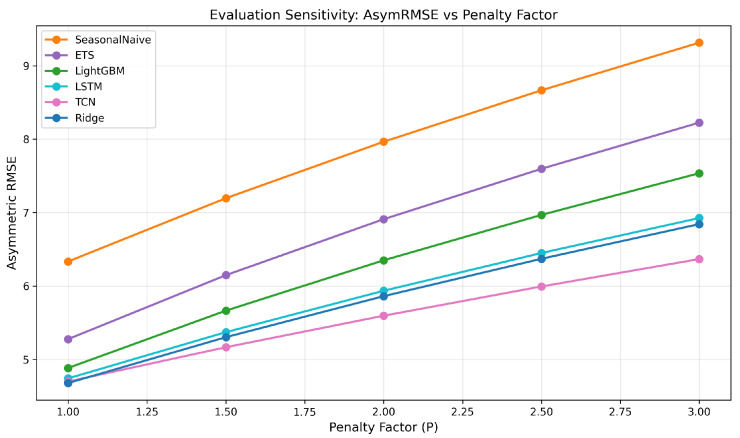
Asymmetric RMSE as a function of the penalty factor *p* for all six models. TCN (pink) achieves the lowest AsymRMSE for all p≥1.5; at p=1.0 TCN and Ridge are effectively tied (Ridge marginally lower). Ridge (blue) sits closest to TCN, with LSTM (cyan) slightly above; the three top-tier models remain visually clustered across the full range. LightGBM, ETS, and Seasonal Naive trail further above. The relative ordering of the top three models is preserved across all penalty values, confirming that the choice of p=2.0 does not bias the comparison.

At the evaluation level, the relative ordering of the top-tier models (Ridge, TCN, and LSTM) is stable across all *P* values, with TCN consistently achieving the lowest AsymRMSE because its asymmetric training has nudged it toward overprediction. At the training level, Ridge’s performance is invariant to the training penalty (MAE = 3.75 for all *p*), as the closed-form L2 solution absorbs the asymmetric weighting through the HPO objective alone. LightGBM shows modest variation (MAE range: 4.00–4.16) that does not change the primary finding that the top-tier ML models perform comparably. These results confirm that the choice of p=2.0 does not drive the reported conclusions. Figure 2 illustrates how AsymRMSE varies with the penalty factor for each model, showing that the curves do not cross in a way that would alter the primary findings.

### 4.4. Feature Ablation Analysis

To assess whether the 183-feature set introduces overfitting risk and to quantify the contribution of each feature group, we conducted a systematic ablation study with 10 configurations across Ridge and LightGBM. Table 7 summarizes the key results for Ridge.

Three findings are notable. First, removing structured autoregressive lags causes the largest degradation (MAE: 3.75 → 4.71), confirming that the daily lookback structure is the most valuable feature group. Second, using only the top-50 features by Ridge importance nearly matches the full model (MAE: 3.79 vs. 3.75), indicating that the additional features do not inflate performance through overfitting. Third, extending the lag window from 7 to 30 days yields progressive improvement (MAE: 3.88 → 3.75), justifying the 30-day design choice. LightGBM exhibits similar patterns across all configurations.

### 4.5. Expanding-Window Cross-Validation

To address the concern that model performance may be sensitive to the specific 14-day test period, we conducted an expanding-window evaluation using three independent month-long test sets covering September, October, and November 2024. In each window the training set is extended up to the start of the test month and the model is retrained from scratch using the same hyperparameters selected for the main run; only the training data, not the hyperparameters, expands across windows.

Both models retain a similar overall accuracy across the three windows, with no systematic monotonic drift over time (Table 8). Ridge MAE varies between 3.32 (October) and 3.79 (November), a 14% range that is consistent with the higher mean arrival rate of the November test period (17.1 patients/hour, versus 13.2 in the full dataset) rather than with model degradation per se. The November window contains the original 14-day hold-out test period (17–30 November) as a subset, and the 30-day November Ridge MAE of 3.79 is in close agreement with the primary 14-day test value of 3.75, confirming that the main hold-out result is representative of the broader month.

Across windows, Ridge is also visibly more stable than LightGBM: LightGBM degrades from 3.31 (October) to 4.17 (November), a 26% increase, whereas Ridge moves only from 3.32 to 3.79 (14%). This larger sensitivity of LightGBM to the harder November period is consistent with the main-run finding that Ridge is marginally significantly better than LightGBM on the primary test set (p=0.028) and provides additional evidence that the linear model generalizes more robustly to shifts in the underlying arrival distribution.

### 4.6. Uncertainty Quantification: Prediction Intervals

To provide probabilistic forecasts, we constructed conformal prediction intervals for the Ridge model. Using the last 14 days of training data as a calibration set, we computed nonconformity scores and applied the conformal quantile method to generate intervals at the 90% and 95% confidence levels.

**Figure 3:**
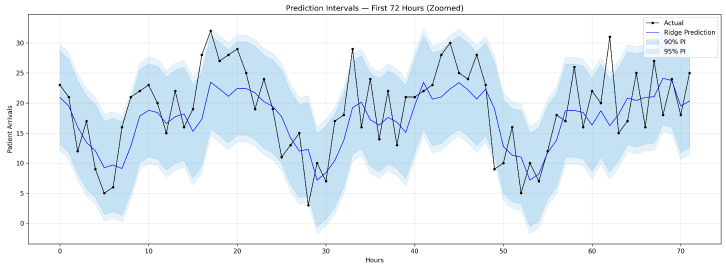
Ridge Regression predictions (solid line) with 90% and 95% conformal prediction intervals (shaded bands) over the first 72 h of the test period. Actual arrival counts (dots) fall within the 90% interval in 91.4% of hours, closely matching the nominal coverage level.

The 90% prediction interval achieved 91.4% empirical coverage (target: 90%) with an average width of 15.5 patients, while the 95% interval achieved 94.0% coverage (target: 95%) with an average width of 17.8 patients. Both intervals are well-calibrated, with coverage rates closely matching nominal levels. Figure 3 illustrates the Ridge predictions with shaded 90% and 95% intervals over a 72 h segment of the test set. These intervals enable operational decision-makers to assess not only the expected arrival rate but also the plausible range of demand for each hour, directly supporting risk-adjusted staffing decisions.

### 4.7. Temporal Analysis and Model Behavior

**Figure 4:**
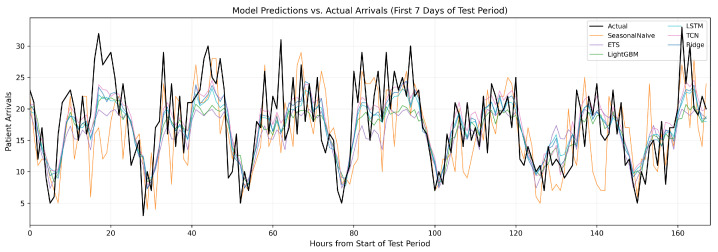
Hourly predictions from all six models overlaid with actual arrival counts (black solid line) during the first seven days of the test period (17–23 November 2024). Ridge, TCN, and LSTM track the dominant diurnal pattern most closely and are visually clustered, consistent with their statistical equivalence on Diebold–Mariano tests. LightGBM produces a similar but slightly smoother trajectory. ETS produces visibly smoother forecasts that lag during sharp transitions, and the Seasonal Naive baseline exhibits characteristic one-week lag artifacts when the current week’s pattern deviates from the previous week. Refer to the in-plot legend for model-to-color mapping.

Figure 4 illustrates the temporal evolution of predictions across the test period. All ML models successfully capture the dominant diurnal and weekly patterns. Ridge Regression and the deep learning models (TCN and LSTM) exhibit stable tracking behavior, while ETS produces visibly smoother forecasts that lag during sharp transitions. Error analysis reveals that accuracy is highest during routine periods and degrades during high-volatility transitions (weekend–weekday shifts, holidays). No systematic degradation is observed over the 14-day horizon, indicating that the models maintain predictive power without frequent retraining.

### 4.8. Model Interpretability: Feature Importance

**Figure 5:**
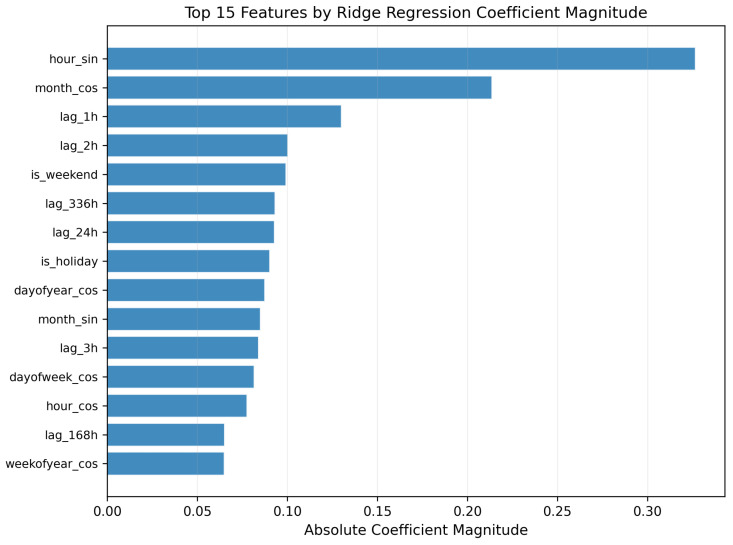
Top 15 features identified by the Ridge Regression model, ranked by absolute coefficient magnitude. Temporal cyclical features (sine and cosine transforms of hour, day of week, and month) dominate the rankings, followed by autoregressive lag features and contextual indicators for weekends and public holidays.

Figure 5 presents the top 15 features identified by Ridge through coefficient magnitude. Temporal cyclical features dominate the ranking: hour_sin, month_cos, month_sin, dayofyear_cos, dayofweek_cos, hour_cos, and weekofyear_cos all appear in the top 15, confirming the strong periodic structure of hourly arrivals. Among autoregressive features, the immediate 1–3 h lags rank near the top, alongside the 24 h, 168 h (one week), and 336 h (two week) lags that encode the dominant daily and weekly recurrence. Weekend (is_weekend) and holiday (is_holiday) indicators also appear among the top features. The balanced distribution across groups, consistent with the ablation analysis, indicates that the model draws on complementary information rather than relying on a single pattern.

### 4.9. Operational Classification Performance

While MAE, RMSE, and AsymRMSE quantify the magnitude of forecast errors, ED staffing decisions are ultimately discrete: shifts are sized for low, medium, or high expected demand. To assess how well each model would support such decisions, we discretized the continuous forecasts into three operational bands using the 33rd and 67th percentiles of the observed hourly arrival counts in the test period as cut-points. Applying the same thresholds to the ground-truth series and to each model’s predictions yielded paired categorical labels, from which we computed a standard multiclass confusion matrix per model. This evaluation is aligned with the actual decision the forecast informs (whether to schedule a low, medium, or high staffing level for the next hour) rather than with pointwise numerical accuracy.

**Figure 6:**
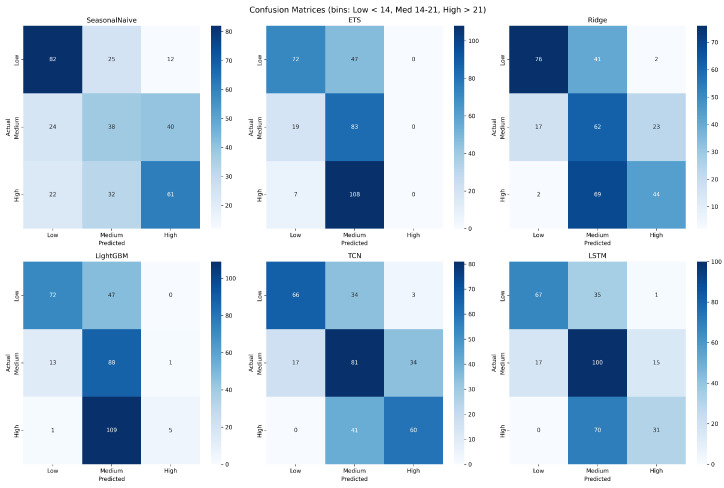
Binned confusion matrices for all six models on the hold-out test period, obtained by discretizing continuous hourly forecasts into low-, medium-, and high-demand bands using the 33rd and 67th percentiles of the observed test-set arrival counts as cut-points. Diagonal cells indicate correct tercile classification; off-diagonal cells indicate misclassification direction. TCN and LSTM achieve the highest overall accuracy (61.6% and 58.9%, respectively) and, crucially, retain meaningful recall on the high-demand tercile (59.4% and 30.7%), whereas LightGBM and ETS concentrate predictions in the medium band and systematically under-detect high-demand hours.

Figure 6 presents the binned confusion matrices for all six models. TCN achieves the highest overall tercile-classification accuracy at 61.6%, followed by LSTM (58.9%), Ridge (54.2%), Seasonal Naive (53.9%), LightGBM (49.1%), and ETS (46.1%). Two observations are notable. First, the operational ranking differs from the pure-MAE ranking: although Ridge, TCN, and LSTM are statistically indistinguishable on point-forecast MAE, the deep sequence models convert their marginal accuracy advantage into a meaningfully larger gain on the high-demand tercile, which is the most operationally consequential class. TCN attains 59.4% recall on the high-demand band, compared with 38.3% for Ridge and only 4.3% for LightGBM. Second, models that minimize squared error without explicit class awareness (LightGBM, ETS) concentrate their predictions in the medium band and systematically under-detect high-demand hours: LightGBM reaches 86.3% recall in the medium band but collapses on the high band, and ETS fails entirely on the high tercile (0.0% recall). This illustrates why point-forecast metrics alone are insufficient for operational evaluation and reinforces the role of the asymmetric loss in pushing the top-tier models, particularly TCN, toward safer high-demand detection.

## 5. Discussion

### 5.1. The Top-Tier Cluster: Ridge, TCN, and LSTM

Ridge Regression, the hybrid TCN, and the hybrid LSTM are statistically indistinguishable from one another on point-forecast MAE (Diebold–Mariano p>0.05 for all three pairwise comparisons). When the engineered feature set explicitly encodes the dominant non-linear structure (cyclical sine/cosine transforms, structured multi-day lags, weekend and holiday indicators, and volatility measures), a regularized linear model matches hybrid sequence models that combine raw arrival sequences with the same static features. Ridge’s L2 regularization provides effective generalization on this moderately sized training set (∼7000 hourly observations), and the closed-form solution removes the variance introduced by stochastic optimization. TCN and LSTM extract additional structure from the raw arrival sequence, but the marginal benefit they provide on point MAE is small enough to be statistically undetectable on a 14-day test set. On the asymmetric metric, however, TCN clearly leads (AsymRMSE 5.59 vs. Ridge 5.86 and LSTM 5.93), reflecting the model’s tendency to err on the safe side of staffing decisions.

LightGBM placed fourth (MAE 3.94), marginally worse than Ridge on the Diebold–Mariano test (p=0.028), though its differences with TCN and LSTM are not statistically significant. Despite its strong reputation on tabular data, gradient boosting did not exceed the performance of either the linear baseline or the deep sequence models on this dataset. The selected hyperparameters favored a relatively shallow ensemble (max_depth = 8, min_child_samples = 95), suggesting that the feature space is already well aligned with linear combinations and that the extra flexibility of tree-based splits offers little additional signal. This is consistent with Vollmer et al. [6], who observed that linear models frequently match or outperform complex ML architectures for ED demand forecasting.

### 5.2. Value of Feature Engineering over Pure Time-Series Methods

The inclusion of ETS allows a direct comparison with a classical state-space method. ETS achieves an MAE of 4.21, meaningfully better than the Seasonal Naive baseline (4.89) but trailing all four feature-engineered ML models, falling roughly 11% short of Ridge. A key difference is access to the engineered feature set: contextual indicators (holidays and weekends), structured multi-day lags, and volatility measures are unavailable to ETS by design. These results suggest that the feature engineering strategy contributes meaningfully to performance beyond what temporal autocorrelation alone can provide.

### 5.3. Generalizability Considerations

This study uses data from a single Emergency Department in Madinah, Saudi Arabia, which introduces several generalizability considerations. The Saudi weekend structure (Friday–Saturday) and Islamic holiday calendar produce arrival patterns that differ from Western hospital systems. While the specific feature values (e.g., holiday dates and weekend days) are site specific, the methodology (cyclical encoding, structured lags, contextual indicators, and asymmetric loss optimization) is generalizable. The expanding-window evaluation demonstrates temporal stability across three independent monthly test periods, providing evidence that the approach is not overfit to a specific time window. However, validation on external datasets from different hospitals, regions, and healthcare systems remains necessary to establish broader applicability. The growing body of Saudi Arabian healthcare ML research [8,9] demonstrates both the feasibility and the distinctive challenges of deploying ML in this context, including pilgrimage-driven demand surges and culturally specific temporal patterns. Multi-site validation across Saudi hospitals with different patient populations and proximity to pilgrimage sites would be a natural next step.

More concretely, the methodological framework (cyclical encodings, structured lags, asymmetric loss, two-phase training, and conformal intervals) is portable to any ED that maintains timestamped registration logs; only the site-specific parameter values (calendar, weekend days, and penalty *p*) need re-derivation. That such a framework transfers well is supported by Vollmer et al. [6] across two London hospitals and by Porto and Fogliatto [18] across 11 EDs in three countries. However, Saudi EDs near major pilgrimage sites experience Hajj- and Umrah-driven surges absent from Western datasets, so the direct transfer of Western-trained models without local recalibration is inadvisable, reinforcing the need for multi-site validation.

### 5.4. Operational Contextualization

Ridge’s MAE of 3.75 patients per hour (TCN: 3.80; LSTM: 3.85) represents the average hourly forecast deviation among the top-tier models. Contextualized against the mean arrival rate of 13.2 patients per hour in the full dataset (and 17.1 per hour in the hold-out test period), this corresponds to a relative error of approximately 21.9% in the test period. Whether this level of accuracy is operationally acceptable depends on site-specific factors such as staffing flexibility, patient acuity mix, and institutional risk tolerance, a determination that requires clinical validation beyond the scope of this study. We note, however, that the conformal prediction intervals provide a principled mechanism for translating point forecasts into risk-adjusted staffing decisions: the 90% interval width of 15.5 patients gives operational planners a concrete range within which to set staffing levels, and the well-calibrated coverage (91.4%) means this range can be trusted for prospective planning.

The practical significance of these gaps depends on the operational context. The 0.05 MAE difference between Ridge and TCN amounts to roughly half a patient over a 10 h shift; because these top-tier models are statistically indistinguishable on the Diebold–Mariano test, the choice between them should be guided by operational priorities: Ridge for interpretability and simple deployment, TCN when under-staffing risk dominates (AsymRMSE 5.59 vs. 5.86; high-tercile recall 59.4% vs. 38.3%; Section 4.9). Larger gaps compound across shifts: the cumulative gap between ETS and the best ML models reaches roughly 5–6 patient-hours of underpredicted demand over a 12 h high-demand period, which is more likely to affect shift-level staffing. Whether any absolute error level is operationally acceptable remains site-specific and requires clinical validation.

### 5.5. Limitations

Several limitations should be acknowledged:

1. **Single-center data:** The study uses data from one ED, limiting external validity. As discussed in Section 5.3, the methodology is portable across sites but the specific feature values are not. Multi-site validation is needed to confirm generalizability across different hospital systems and geographic regions.
2. **Statistical power of between-model tests:** The hold-out test set contains 336 h, which limits the power of the Diebold–Mariano comparisons. Differences smaller than approximately 0.10–0.15 in MAE may not be reliably detected on a window of this length, and the “statistically indistinguishable” verdict for the Ridge/TCN/LSTM cluster should be interpreted accordingly. The expanding-window evaluation in Section 4.5 partially mitigates this by replicating the comparison across three additional monthly periods.
3. **Limited observation window:** The 11-month dataset may not capture full annual cyclicality (e.g., Hajj pilgrimage effects and winter respiratory seasons). A full 12+ month dataset would strengthen the seasonal feature analysis.
4. **No external contextual features:** Weather data, nearby event schedules, and epidemiological indicators were not available for this study but could improve performance.
5. **No domain expert validation:** The operational interpretation of forecast errors has not been validated through formal consultation with ED staff or management. Future work should involve clinical stakeholders in defining acceptable error thresholds.
6. **Deterministic feature engineering:** The feature set is manually designed. Automated feature selection or learned representations may improve performance on larger datasets.

## 6. Conclusions

This study develops and evaluates a unified framework for hourly Emergency Department arrival forecasting, comparing six models spanning a classical baseline (Seasonal Naive), a state-space method (ETS), statistical learning (Ridge and LightGBM), and two hybrid deep learning architectures (TCN and LSTM). Using real data from an ED in Madinah, Saudi Arabia, and a feature set of 183 engineered predictors verified to be free of data leakage, we find that Ridge Regression delivers the lowest MAE (3.75, R^2^ = 0.52), with TCN (3.80) and LSTM (3.85) being statistically indistinguishable from Ridge on Diebold–Mariano tests. TCN achieves the lowest Asymmetric RMSE (5.59), reflecting its tendency to err on the safe side of staffing decisions. Ridge is marginally significantly better than LightGBM (p=0.028); all four ML models significantly outperform ETS and the Seasonal Naive baseline.

These findings are supported by: (1) a sensitivity analysis confirming stable model rankings across penalty factors p∈[1.0,3.0]; (2) a feature ablation study demonstrating that each feature group contributes to performance without overfitting; (3) expanding-window cross-validation showing consistent results across three independent monthly test periods; and (4) conformal prediction intervals achieving well-calibrated coverage (91.4% at the 90% nominal level).

The central practical finding is that, when paired with a careful feature engineering pipeline, a lightweight linear model matches hybrid deep learning architectures and outperforms gradient-boosted trees and classical statistical baselines on this task. Ridge offers operational advantages in interpretability, computational efficiency, and ease of deployment, while TCN provides a slight advantage when underprediction must be penalized more heavily. Future work should pursue multi-hospital validation, incorporate additional contextual features (weather, epidemiological indicators), and engage clinical stakeholders in defining operational decision rules based on the probabilistic forecasts.

## Figures and Tables

**Table 1:** Positioning of the present study relative to key comparable works in ED arrival forecasting.

Study	Year	Granularity	Models Compared	Feature Eng.	Asym. Loss	Region
Vollmer et al. [6]	2021	Daily	GLM, RF, GB, kNN	Calendar	No	UK (2 hospitals)
Rostami-Tabar et al. [2]	2024	Hourly	GAM, ES, Prophet	None (univariate)	No	Wales
Tuominen et al. [7]	2024	Daily (24 h)	LightGBM, DeepAR, TFT	EHR, traffic, weather	No	Finland
Porto and Fogliatto [18]	2024	Daily	XGBoost, LightGBM, SVM, NNAR	Calendar, meteorological	No	Multi-site (3 countries)
**This study**	**2026**	**Hourly**	**ETS, Ridge, LightGBM,** **TCN, LSTM + Seasonal Naive**	**183 features (temporal, contextual, autoregressive)**	**Yes**	**Saudi Arabia**

**Table 2:** Experimental data specification, feature composition, and chronological partitioning.

Component	Description	Details/Dimensions
**I. Dataset Specification**		
Raw Data Period	De-identified, timestamped ED registration records from a single center.	1 January–30 November 2024
Target Variable (Y)	The total number of patient arrivals per one-hour interval.	Integer, Hourly Freq.
**II. Predictor Variable Composition (X): Total 183 Features**		
**Temporal and Cyclical**	Sine/cosine transforms of hour, day of week, day of month, and month to represent periodicity.	10 features
**Event-Based and Contextual**	Binary indicators for weekends (Friday/Saturday), and official public holidays in Saudi Arabia.	2 features
**Autoregressive**	Structured lags: immediate momentum (1–3 h prior) and daily context (a 4 h window on each of the preceding 30 days).	123 features
**Volatility**	First- and second-order differences of the target variable, with lags up to 24 h prior, to capture rate of change.	48 features
**III. Experimental Design: Data Partitioning**		
Training Set	Data used for model training and hyperparameter optimization.	1 January–16 November 2024 (∼6981 samples)
Test Set (Hold-out)	Unseen data from the final 14 days, reserved for unbiased performance evaluation.	17 November–30 November 2024 (336 samples)

Note: Approximately 9% of raw hourly observations (723 samples) were lost during feature engineering due to NaN values from lagged features.

**Table 3:** Hyperparameter optimization configuration and search spaces for the AMSE objective.

Hyperparameter	Description	Search Space and Distribution
**Ridge Regression**		
Regularization Strength (α)	Controls the strength of L2 regularization to prevent overfitting.	Log-uniform from $10^{-2}$ to $10^{2}$
**LightGBM**		
Number of Estimators	The total number of boosting rounds or decision trees to build.	Integer from 200 to 3000
Learning Rate	Step-size shrinkage to prevent overfitting; lower values require more estimators.	Log-uniform from $5\times 10^{-3}$ to $10^{-1}$
Number of Leaves	Maximum number of leaves in a single tree; a key parameter for model complexity.	Integer from 20 to 150
Maximum Tree Depth	Maximum depth of an individual tree; limits model complexity.	Integer from 5 to 20
Minimum Child Samples	Minimum number of data points required in a leaf node.	Integer from 10 to 100
Subsample Ratio	Fraction of data samples to be used for fitting individual trees (row sampling).	Uniform from 0.6 to 1.0
Column Sample Ratio	Fraction of features to be used for fitting individual trees (column sampling).	Uniform from 0.4 to 1.0
**Temporal Convolutional Network (TCN)**		
Number of Filters	The number of convolutional filters in each TCN layer.	Integer from 16 to 64 (step 8)
Kernel Size	The size of the convolutional kernel.	Categorical choice from {2, 3, 5, 7}
Dilation Factors	A list of factors controlling the exponential growth of the receptive field.	Categorical choice from {[1, 2, 4, 8], [1, 2, 4, 8, 16]}
TCN Dropout Rate	Dropout rate within the TCN blocks to prevent overfitting on sequential features.	Uniform from 0.0 to 0.4
Dense Layer Dropout	Dropout rate in the final dense layers before the output prediction.	Uniform from 0.1 to 0.5
Learning Rate	The learning rate for the Adam optimizer.	Log-uniform from $5\times 10^{-4}$ to $10^{-2}$
**Long Short-Term Memory (LSTM)**		
LSTM Units	Number of hidden units in each LSTM layer.	Integer from 16 to 128 (step 16)
Number of Layers	Number of stacked LSTM layers.	Categorical choice from {1, 2}
LSTM Dropout Rate	Dropout rate within the recurrent cells.	Uniform from 0.0 to 0.4
Dense Layer Dropout	Dropout rate in the final dense layers before the output prediction.	Uniform from 0.1 to 0.5
Learning Rate	The learning rate for the Adam optimizer.	Log-uniform from $5\times 10^{-4}$ to $10^{-2}$

**Table 4:** Best hyperparameters identified by Bayesian optimization.

Model	Hyperparameter	Optimal Value
Ridge	Regularization Strength (α)	99.59
LightGBM	Number of Estimators	2100
	Learning Rate	0.0670
	Number of Leaves	105
	Maximum Tree Depth	8
	Minimum Child Samples	95
	Subsample Ratio	0.865
	Column Sample Ratio	0.487
TCN	Number of Filters	16
	Kernel Size	2
	Dilation Factors	[1, 2, 4, 8, 16]
	TCN Dropout Rate	0.265
	Dense Layer Dropout	0.146
	Learning Rate	0.00240
LSTM	LSTM Units	48
	Number of Layers	1
	LSTM Dropout Rate	0.341
	Dense Layer Dropout	0.126
	Learning Rate	0.00631

**Table 5:** Comprehensive performance metrics on test set (14 days).

Metric	Ridge	TCN	LSTM	LightGBM	ETS	S.Naive
MAE	**3.75**	3.80	3.85	3.94	4.21	4.89
RMSE	**4.68**	4.70	4.74	4.88	5.28	6.33
R^2^	**0.516**	0.512	0.503	0.473	0.385	0.113
AsymRMSE	5.86	**5.59**	5.93	6.35	6.91	7.96
AUCt	3.96	**3.65**	4.02	4.51	4.68	5.76
Underpredict %	50.3	43.8	50.3	54.5	58.9	44.3
Overpredict %	49.7	56.2	49.7	45.5	41.1	48.8

Bold = best. AsymRMSE uses penalty *p* = 2.0 for underpredictions. Percentages may not sum to 100% due to exact predictions (zero residual).

**Table 6:** Diebold–Mariano test: pairwise comparisons.

Model 1	Model 2	DM Stat.	Result
S.Naive	Ridge	5.77	Ridge better ***
S.Naive	TCN	5.56	TCN better ***
S.Naive	LSTM	5.55	LSTM better ***
S.Naive	LightGBM	4.73	LightGBM better ***
S.Naive	ETS	3.53	ETS better ***
ETS	Ridge	4.32	Ridge better ***
ETS	TCN	3.27	TCN better **
ETS	LSTM	4.37	LSTM better ***
ETS	LightGBM	3.78	LightGBM better ***
Ridge	LightGBM	−2.20	Ridge better *
Ridge	TCN	−0.31	Not significant
Ridge	LSTM	−1.84	Not significant
LightGBM	TCN	1.43	Not significant
LightGBM	LSTM	1.64	Not significant
TCN	LSTM	−0.59	Not significant

* p<0.05, ** p<0.01, *** p<0.001.

**Table 7:** Feature ablation study (Ridge Regression).

Configuration	Features	MAE
Full model	183	3.75
Top-50 by importance	50	3.79
14-day lag window	119	3.82
7-day lag window	91	3.88
Full-Volatility	135	3.77
Full-Cyclical	173	3.75
Full-Event	181	3.75
Immediate + Cyclical + Event	15	4.54
Full-Structured Lags	60	4.71
Immediate lags only (1–3 h)	3	4.77

**Table 8:** Expanding-window validation results.

Test Period	Test Size	Ridge MAE	LightGBM MAE
September 2024	720	3.46	3.47
October 2024	744	3.32	3.31
November 2024	720	3.79	4.17

## Data Availability

The data presented in this study are available from the corresponding author upon reasonable request. The data are not publicly available due to patient confidentiality. To facilitate the reproduction of the results, the algorithm code is available at https://github.com/mah-sam/ed-arrival-forecasting (accessed on 21 April 2026).
